# Riedel’s thyroiditis: clinical presentation, treatment and outcomes

**DOI:** 10.1007/s12020-018-1526-3

**Published:** 2018-01-29

**Authors:** Henrik Falhammar, Carl Christofer Juhlin, Caroline Barner, Sergiu-Bogdan Catrina, Christos Karefylakis, Jan Calissendorff

**Affiliations:** 1 Department of Endocrinology, Metabolism and Diabetes, Karolinska University Hospital, Stockholm, Sweden; 2 Department of Molecular Medicine and Surgery, Karolinska Institutet, Stockholm, Sweden; 3 Department of Clinical Pathology and Cytology, Karolinska University Hospital, Stockholm, Sweden; 4 Department of Oncology-Pathology, Karolinska Institutet, Stockholm, Sweden; 5 Department of Medicine, Capio S:t Gorans Hospital, Stockholm, Sweden; 6 Department of Diabetes, Endocrinology and Metabolism, School of Medical Sciences, Örebro University, Örebro, Sweden

**Keywords:** Riedel, Inflammation, IgG4, Diagnose, Glucocorticoid treatment

## Abstract

**Background:**

Riedel´s thyroiditis (RT) is a rare inflammatory disease of the thyroid gland, causing compression and fibrosis of adjacent tissues. Typically the goiter is hard and firm. Hoarseness, dyspnea, and dysphagia may be present.

**Methods:**

We retrospectively reviewed all patients known by us with RT in addition to all patients with appropriate ICD-10 codes evaluated at the Karolinska University Hospital 2003–2015. Clinical, biochemical, and histological data of patients with RT were recorded in detail. Histological preparations were re-examined when available.

**Results:**

RT was diagnosed in six patients. Five were females and the median age at first presentation was 50 years (25–81 years). Median follow-up time was 3.75 years (1–22 years). At diagnosis five had hypothyroidism. Four had extrathyroidal manifestations, and one of these had also distant fibrosis. One patient had a clear IgG4/IgG ratio over 40%. One patient was treated with tracheostomy, one with isthmectomy and one with total thyroidectomy. Four had been treated with glucocorticoids, four with tamoxifen, and two with both drugs. One had also been treated with mycophenolate mofetil combined with Rituximab. At the end of follow-up four was doing fine, one had recurrent episodes of inflammation and one had died of possible complications to RT.

**Conclusion:**

It is important to recognize RT and give adequate treatment. Steroids are still the mainstay of therapy but other medications against fibrosclerosis can be considered. Wakefulness of other fibrosing manifestations is essential. Immunohistochemistry can show whether IgG-4 plasma cells are increased which could lead to fibrosis in other organs.

## Introduction

Riedel’s thyroiditis (fibrous thyroiditis, RT) is an extremely rare form of thyroiditis, leading to a gradual parenchymal transformation to connective tissue. Clinically this is a hard, palpable goiter, which could cause pain, compressive symptoms, and affect adjacent structures as the parathyroid glands, musculature in the neck and cause vocal cord paralysis. The etiology is not completely understood, but inflammation mediated by mononuclear blood cells appears to be of importance. RT has been claimed to be an IgG4-related disease, also termed IgG4-related thyroid disease [1]. This disease group includes orbital pseudotumors, interstitial pneumonia, autoimmune pancreatitis and RT [2].

In RT, fibrosclerotic organ manifestations can either be a part of IgG4-related disease or solely be located in the thyroid and adjacent tissues. Elevation of serum IgG4 can be found, but more often lymphoplasmocytic IgG4-positive plasma cells and storiform fibrosis are present in thyroid tissue. The ratio of thyroid infiltrating IgG4/S-IgG4 in plasma >40% has been used as part of the diagnostic work-up. It can be difficult to separate the fibrosing variant of Hashimoto’s thyroiditis from IgG4-related thyroid disease [3, 4]. Hürthle cell metaplasia and an enhanced Doppler flow on ultrasound suggests fibrosing Hashimoto, while extrathyroidal engagement (usually adjacent strap muscle) is present in RT. Giant cells (as seen in de Quervain thyroiditis) must not be present. Differentiation between RT and other inflammatory conditions has been further characterized [5]. The net effect of RT is a loss or compression of the normal thyroid follicular structure, a pronounced fibrotisation of the thyroid gland and sometimes also of adjacent structures such as the parathyroid, trachea, and mediastinum [6]. Patients can present with a slowly growing non-tender goiter of varying size, which at palpation is hard and firm (“iron hard”). Hoarseness, dyspnea and dysphagia may be present. Most patients are euthyroid but not all. Treatment is empirical with long-term treatment with glucocorticoids, to decrease the inflammation being first choice [5], but tamoxifen has also been used [7]. The mechanism of tamoxifen is not known but is thought to be mediated by inhibition of fibroblast proliferation [8]. Rituximab has been shown to be of use in a patient refractory to glucocorticoids and tamoxifen [9]. Surgery is indicated to relieve obstruction but is associated with risks of damage of the recurrentlaryngeal nerve and hypoparathyroidism.

The aims of the present study were to report our experience of RT.

## Methods

All cases with RT treated by us between 1997 and 2016 were retrospectively reviewed. Moreover, the electronic medical files of all patients seen at the Department of Endocrinology, Metabolism and Diabetes, Karolinska University Hospital, Stockholm, Sweden, between 2003 and 2015, with an International Classification of Diseases version 10 (ICD-10) code of E06.5 (Other chronic thyroiditis, according to WHO the one applicable to RT), E06.9 (Thyroiditis, unspecified), E07.1 (Dyshormogenetic goiter), E07.8 (Other specific disorders of thyroid) and E07.9 (Disorder of thyroid, unspecific) were reviewed to investigate if additional cases could be found. All specialist outpatient visits and admissions in Sweden are coded with ICD-10 codes by the attending physician and are thereafter stored in both local and national databases [10]. The clinical, biochemical, and histological data of patients with RT were recorded in detail. If available, the histological preparations were reviewed by CCJ.

The study was in accordance with the ethical standards of the institutional, national research committee and with the 1964 Helsinki declaration and its later amendments or comparable ethical standards.

## Results

Six patients with RT were already known and no additional cases were identified using the ICD-10 codes. Thus, six cases were included, five females (83%) and one male with a median age at first presentation of 50 years (25–81 years). Median follow-up time was 3.75 years (1–22 years). Four had extrathyroidal fibrosis, and one of these had also distant fibrotic manifestations. Four were treated with glucocorticoids, two of those also with tamoxifen, and two with only tamoxifen. One had first been treated with glucocorticoids and later with mycophenolate mofetil combined with rituximab. Three patients underwent surgery; one was treated with tracheostomy, one with isthmectomy and one with total thyroidectomy. Fine needle aspiration (FNA) was performed in five patients and excision biopsy in one patient. One patient had a clear IgG4/IgG ratio over 40%. An overview of all cases is presented in Tables 1–4 and details of each case are reported below.

**Table 1:** Characteristics and follow-up time in six cases of Riedel’s thyroiditis

Case	Age (years)	Smoker	Ethnicity	Hypothyroidism	Follow-up (years)
1 F	81	No	Caucasian	No	4.5
2 F	47	Former	Caucasian	Yes	18
3 F	55	No	Arab	Yes	1
4 F	53	Yes	African	Yes	1.5
5 M	32	No	Caucasian	Yes	3
6 F	25	Yes	Caucasian	Yes	22
Median	50				3.75

*F* female, *M* male

**Table 2:** Initial laboratory and immunohistochemistry in Riedel’s thyroiditis

Case	TSH	Free T4	Free T3	TPOab	Tgab	TRAb	Calcium	PTH	CRP
1	0.01	26	8.1	Neg	Neg	Neg	Normal	Normal	142
2	Elevated	Low	NA	NA	NA	NA	Normal	Normal	Elevated
3	123	9	4.4	Neg	Neg	Neg	2.29	Normal	Normal
4	19	5	2.4	Neg	Pos	Neg	1.21*	<0.3	Elevated
5	118	<5.5	<1.5	Pos	NA	Neg	Normal	Normal	10
6	111	2	2.7	Pos	Pos	Pos	Normal	Normal	119

Normal range: TSH 0.3–4.2 mIU/L; free T4 12–22 pmol/L; free T3 3.1–6.8 pmol/L; thyroid peroxidase antibodies (TPOab) <34 kIU/L; thyreoglobulin autoantibodies (Tgab) <4.0 kIE/L; thyroid receptor antibodies (TRAb) <1.8 mIU/L; calcium 2.15–2.50 mmol/L; PTH 1.5–7.6 pmol/L; CRP <3 mg/L; * ionized calcium 1.15-1.33 mmol/L

*NA* not available

**Table 3:** Time from start of symptoms and type of biopsy, and treatment in Riedel’s thyroiditis

Case	Time to biopsy	Type of biopsy	Steroids	Tamoxifen	MM	Rituximab	Surgery
1	3 months	FNA + surgery	Yes	Yes	No	No	Tracheostomy
2	6 months	Excision	Yes	Yes	No	No	No
3	4 months	FNA	No	Yes	No	No	No
4	2 months	FNA	No	Yes	No	No	No
5	6 months	FNA + surgery	Yes	No	No	No	Isthmectomy
6	4 years	Biopsy	Yes	No	Yes	Yes	Thyroidectomy

*FNA* fine needle aspiration, *MM* mycophenolate mofetil

**Table 4:** IgG4 in serum, in thyroid tissue and % IgG4 with immunohistochemistry in Riedel’s thyroiditis

Case	Histopathology	S-IgG4	Tissue IgG4	Tissue IgG4/IgG
1	Lymphocytes, granulocytes, scarlike fibrosis, and extrathyroideal engagement	NA	Yes	50%
2	Fibrosis, no visible thyroid cells	NA	NA	NA
3	Massive infiltrates of inflammatory cells, extrathyroidal engagement	Neg	NA/FNA	NA/FNA
4	Inflammatory cells, fibroblasts, and no thyroidal tissue	Neg	NA/FNA	NA/FNA
5	Fibrosis, lymphocytes, and extrathyroideal engagement	Neg	Yes	2%
6	Inflammatory cells, extrathyroideal engagement	Neg	Yes	13%

*NA* not available, *FNA* fine needle aspiration

### Case 1

An 81-year-old woman presented to the Emergency Department with dyspnea, coughing and fever for one week. She had lost 9 kg of weight the last 3 months, and had been tired, depressed and had developed a tremor. A non-tender lump was felt on the right side of the thyroid. She had mild hyperthyroidism (Table 2). A radiouptake scan was performed and she was diagnosed with toxic adenoma and commenced on block and replace with methimazole and levothyroxine. Radioiodine treatment was given later and she was continued on levothyroxine 100 mcg daily.

**Fig. 1:**
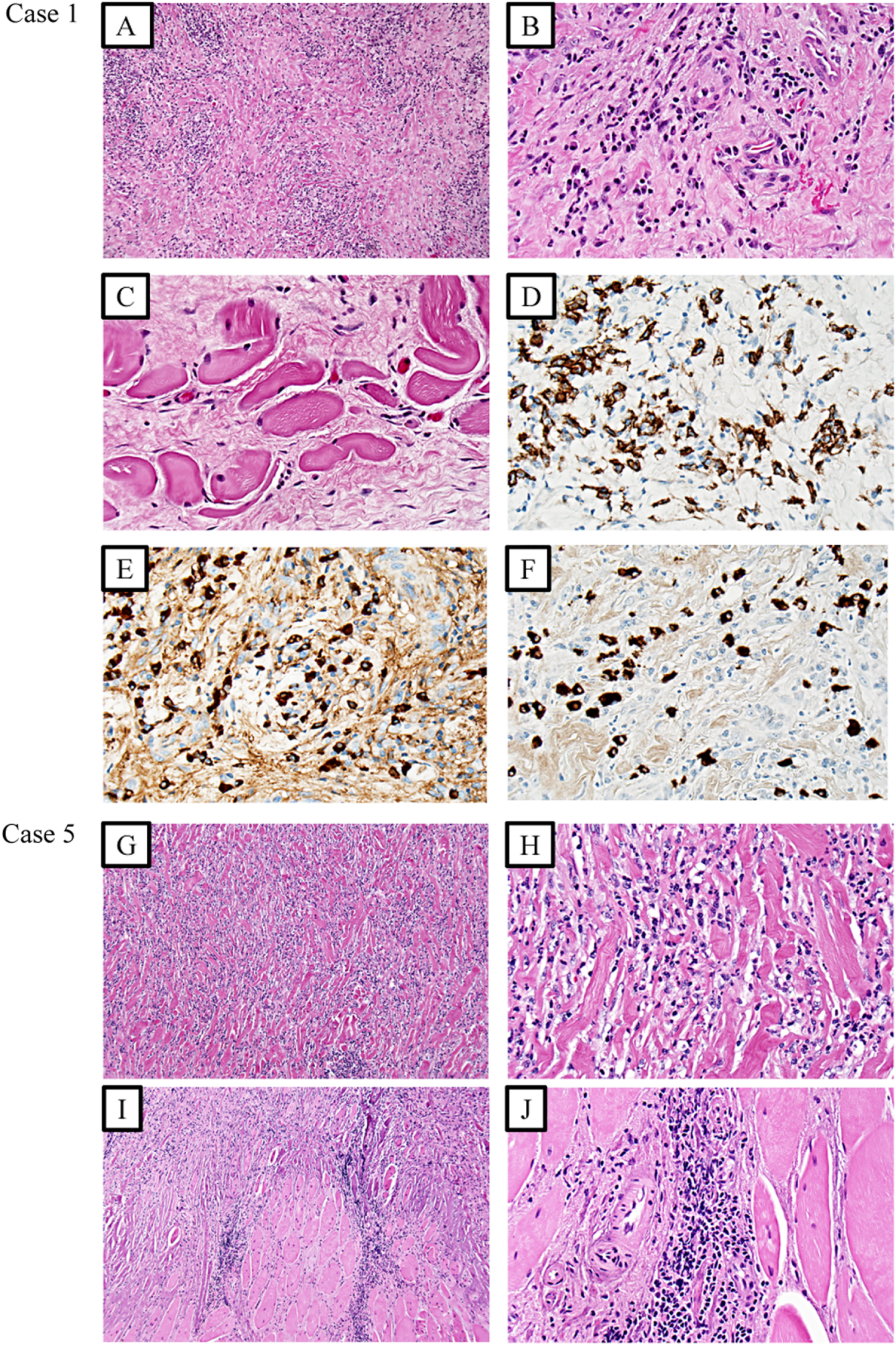
Photomicrographs of case 1 (**a**–**f**) and case 5 (**g**–**j**) with representative features of Riedel´s thyroiditis. All photos except (**d**–**f**) represent haematoxylin-eosin stainings. **a**, **b** Storiform, keloid-like fibrosis, and inflammatory cells have replaced the thyroid parenchyma (magnified x100 and x400 respectively). **c** The inflammatory cells and fibrosis engage the perithyroidal skeletal musculature (x400 magnification). **d** CD138 immunohistochemistry visualizes the infiltrative plasma cells (x400 magnification). **e**, **f** IgG-positive and IgG4-positive cells respectively in the same area, cells at x400 magnification. The number of IgG4-positive cells are >10/high power field, and the IgG4/IgG ratio is >0.5, suggestive of IgG4-related disease. **g**, **h** Storiform, keloid-like fibrosis, and inflammatory cells have replaced the thyroid parenchyma (magnified x100 and x400 respectively). **i**, **j** The inflammatory cells and fibrosis engage the perithyroidal skeletal musculature (x100 and x400 magnification respectively)

Four years later she represented at the Emergency Department with dyspnea, stridor and coughing. During the last 3 months she could only swallow liquid drinks. CT neck showed a 35 × 55 × 30 mm large tumor on the right side of the thyroid severely compressing the trachea. An acute tracheostomy was performed. Three FNAs from the tumor showed fibrous tissue and mononuclear cells consistent with RT. A biopsy was histologically confirmed as RT with inflammation, destruction of thyroid parenchyma, storiform fibrosis, and extrathyroidal extension (Fig. 1). Moreover, an elevated number of IgG4 positive plasma cells were observed, raising the possibility of IgG4-related disease (Fig. 1; Table 4). The patient denied any further surgical intervention and was started on betamethasone 7 mg daily and tamoxifen 20 mg daily. Two weeks later the tumor felt less firm, a slight decrease was seen on the CT neck. She was discharged continuing treatment with betamethasone 4 mg and tamoxifen 20 mg daily. She could now swallow solid food.

The betamethasone dose was slowly decreased to 2 mg daily while tamoxifen was not changed during the following months. Six months after the tracheostomy the nursing home reported that the tumor had grown significantly, but the patient refused to go to hospital for evaluation. Betamethasone was changed to prednisolone 60 mg daily. Seven months after the tracheostomy and 4.5 years after the initial presentation she was found dead in her bed at an age of 85 years.

### Case 2

A 47-year-old female presented with a lump in the neck, lethargy, dyspnea, and occasional dysphagia. Hypothyroidism was diagnosed and she gradually commenced levothyroxine to a stable dose of 200 mcg daily. Excision biopsy was performed to exclude malignancy, and the histology showed thyroid parenchyma replaced with fibrosis consistent with RT. A few weeks of prednisolone was tried with no relief of symptoms.

Seven years later she was referred again with a large, firm goiter. She had dyspnea and was tired. A CT neck demonstrated tracheal narrowing, but no displacement. A review by an endocrine surgeon was performed and it was decided that thyroid surgery would be inappropriate.

One year later there was worsening dyspnea. CT neck showed retrosternal extension and further compression of the trachea. Prednisolone 75 mg was commenced and tapered over the following 2 months with significant improvement of dyspnea and dysphagia. A repeat CT scan indicated a slight reduction of the size of the thyroid gland as well as an increased lumen of the trachea. Once prednisolone was ceased tamoxifen 20 mg daily was commenced but was stopped after only 3 months due to depression, a suspected side effect of the medication. She had subsequent follow-up visits once a year with no worsening of any symptoms.

Sixteen years after first presentation she represented with stridor, dyspnea and intermittent dysphagia. A CT of the neck showed a large thyroid with obstruction of the trachea to 1/3 the expected size. The thyroid was not significantly larger than previous scans. Prednisolone was commenced at a dose of 50 mg daily, which was tapered over the following 2 months. Stridor and dysphagia disappeared and dyspnea improved. She was seen regularly in the clinic and at the last visit she was 65 years old (18 years after first presentation), quite asymptomatic and only on levothyroxine treatment.

### Case 3

A 55-year-old woman with previous hypothyroidism (TSH 123 mIU/L and TPOab negative at diagnosis 5 months earlier, now normalized with levothyroxine) presented with a swelling of the throat, hoarseness, and dysphagia. The thyroid gland was very firm at palpation. At FNA the tissue was glutinous and the aspirate was dominated by inflammatory cells, lymphocytes, macrophages, and plasma cells. There was also fibrosis and degenerative changes, as well as inflammatory infiltration of the skeletal musculature; findings in line with RT. She was started on tamoxifen 20 mg twice daily and within 3 months she was much improved, the thyroid gland was less swollen and not as firm. At that time S-IgG4 was 0.38 g/L (normal 0.05–1.25). Tamoxifen was reduced to 20 mg daily 7 months later, and was withheld after an additional 2 months. At last follow-up, 1 year after initial presentation, the goiter was still easily palpable but the patient was feeling well and experienced no local symptoms.

### Case 4

A 53-year-old woman presented with a goiter. A FNA of this had 4 years earlier been benign with hyperplastic follicle cells rich of cytoplasm arranged in acinar formation. Two years previously hypothyroidism was diagnosed (Table 2). Levothyroxine was initiated and gradually increased to 100 mcg daily. A new biopsy was performed; the gland was enlarged, very hard at palpation and did not move during swallowing. During the FNA the gland was stiff and the material had a thin protein precipitate with collagen fragments and lymphocytic cells, as in RT. She had difficulties in swallowing and a tenderness of the thyroid gland but no dyspnea. S-IgG4 was normal (0.15 g/L, normal 0.05–1.25), whereas IgG 1, 2, and 3 all were slightly elevated (8.49 g/L, normal 2.8–8.0; 6.76 g/L, normal 1.15–5.7; 2.71 g/L, normal 0.24–1.25, respectively). Since she had diabetes, glucocorticoid therapy was avoided and tamoxifen 20 mg twice daily was started. She experienced improvement and tamoxifen was with held after 9 months, without any recurrent symptoms at the last follow-up one and a half year after presentation.

### Case 5

A 32-year-old male diagnosed a few weeks earlier with hypothyroidism and commenced on 25 mcg of levothyroxine presented with extreme tiredness. He had concentration difficulties and his muscles were aching. He had difficulties speaking and was snoring because of tongue enlargement. Thyroid hormone tests showed severe hypothyroidism (Table 2). Levothyroxine dose was increased. At palpation the thyroid lobes were enlarged and isthmus had an irregular structure but normal consistency. A thyroid ultrasound and histopathology from FNA were consistent with a chronic lymphocytic thyroiditis.

Five months later a difference in consistency and further enlargement of the right thyroid lobe was noted. A second FNA of the right thyroid lobe was performed and histopathology was suggestive of chronic lymphocytic thyroiditis. Medication with levothyroxine was stable at 225 mcg daily.

One year later the patient had local symptoms in the neck area, when turning the head to the right side but had no other symptoms. Thyroid ultrasound and FNA was performed with the same results as previously and medication remained unchanged.

A year later he experienced difficulties with breathing especially when physically active. He had inhaled salbutamole without any effect. Upon admission the thyroid was enlarged, very hard at palpation and now tender to the touch. He had an inspiratory stridor and spontaneous muscle fasciculation. Laboratory revealed hypoparathyroidism with PTH < 0.3 pmol/L (normal 1.5–7.6), total calcium 1.20 mmol/L (normal 2.15–2.50), 25-OH-vit D 65 nmol/L (normal 50–178), and hyperphosphataemia 2.0 mmol/L (normal 0.7–1.7). CT neck/thorax showed enlarged thyroid lobes and isthmus with a compromised tracheal lumen of 6 mm. Betamethasone 8 mg iv, calcium gluconate bolus injections, followed by saline infusion and alphacalcidol 0.5 mcg twice daily was initiated. Due to a persistent stridor a subacute isthmectomy was performed 3 days later, which proved to be difficult because of extensive inflammation. Histopathology showed extensive inflammation, fibrosis and extra thyroidal engagement consistent with RT, but no signs of IgG4-related disease (Fig. 1; Table 4). CT scan of the thorax and a MRI of the abdomen showed no abnormalities and S-IgG4 was negative (Table 4). Betamethasone was reduced to 3 mg once daily for 1 month and further reduced to 2.5 mg for a month. Glucocorticoids were tapered and stopped after 11 months. Levothyroxine, alphacalcidol, and calcium carbonate were continued, and he was stable with no signs of inflammatory activity at last follow-up 3 years after initial presentation.

### Case 6

A 25-years-old woman with an autoimmune hypothyroidism since 2 years, treated with levothyroxine 200 mcg daily, was admitted with fever (37.9 ^o^C). She had neck pain and a rapidly growing goiter, mainly affecting the left lobe. Inflammatory parameters were elevated (ESR 140 mm/h, CRP 119 mg/L, normal <20 and <5, respectively). Based on this clinical presentation together with an initial FNA that had showed a purulent material with granulocytes and fibrin, acute thyroiditis was suspected and antibiotics were initiated. Two days later the diagnosis was reevaluated to subacute thyroiditis because of the clinical appearance and a new FNA showing a mixture of histiocytes, lymphocytes, and granulocytes. Prednisolone 40 mg once daily was initiated, and the dose were tapered to 7.5 mg/day, as the volume of the goiter decreased with clinical improvement. After 1 month a clinical deterioration was noted. A third FNA was performed from the right lobe showing granulocytes, eosinophils, and a large number of partially destroyed muscle cells that was judged to be a late phase of a subacute thyroiditis, and the prednisolone dose was increased to 30 mg/day. During the next 4 years, the patient went through different periods of activation of the disease, which responded, well but transitory to glucocorticoid therapy. The FNAs showed every time an inflammatory process with a mixture of granulocytes, histiocytes, plasma cells and fibroblasts and fragments of muscle cells. A biopsy of a lymph node from the neck showed reactive changes with an increase of plasma cells.

After 4 years of evolution total thyroidectomy including extirpation of some lymph nodes was performed, and RT with extension outside the thyroid gland was the final pathological diagnosis.

Eight years after surgery, and 12 years after initial presentation, the patient still had exacerbations of the general and local symptoms that always were treated with prednisolone for different periods of time. Finally a biopsy was taken from the right maxillary sinus secondarily to symptoms related to the sinus. The pathological diagnosis was IgG4 sclerosing disease. No serum increase of IgG4 was ever detected. Azathioprine was initiated but was stopped secondary to an increase of the liver enzymes. During the following 10 years she was treated with mycophenolate mofetil combined with Rituximab because of lack of effect of mycophenolate mofetil alone, and at last follow-up 22 years after the initial presentation she still has periods of inflammation.

## Discussion

In the literature, patients with RT have only been described in case reports, and in two, partly overlapping, case series including 21 and 6 patients, respectively [11, 12]. Thus, the present study describing our experience of RT is one of the larger cohorts of this very rare condition. The diagnosis was not straight forward, as different clinical symptoms, diagnostic procedures such as FNA or biopsy and imaging modalities were performed. There are no clear cut diagnostic criteria for RT, and the full extent of the disease may not be obvious before surgery (to decompress or to rule out malignancy), or imaging such as FDG-PET have been performed.

Cytology is not always diagnostic in RT, as we also showed, and can fail to demonstrate the invasive nature of the fibrosis, which can cause uncertainties whether the condition is a fibrosing variant of Hashimoto’s disease. Core needle biopsies would provide more information, but are generally not advocated due to the discomfort and potential side-effects associated with such a procedure. More often, the diagnosis of RT is suggested after histological assessment of resected thyroid tissue. Findings such as storiform fibrosis and occlusive phlebitis argues in favor of RT, and in cases of IgG4 involv**e**ment elevation of IgG4-positive plasma cells with an IgG-4 ratio >40% [13]. However, extrathyroidal manifestations of the inflammatory activity remain the most reliable criteria for this disease.

Another factor to take into consideration when diagnosing RT from a cytological or histological perspective, is the time from debut of symptoms to the fine needle biopsy/thyroid resection, as well as the current anti-inflammatory treatment of the patient. As both these parameters may affect the degree of inflammation and hence the amount of storiform fibrosis formed, this could ultimately confuse the diagnosing physician. Steroids may in theory disguise the severity of the disease if histology assessment is substantially delayed, but this has not been studied.

Surgery in these patients is used to relieve pressure symptoms, but is not the mainstay of treatment as tissues are fibrotic and the risks of surgical complications as hypoparathyroidism or damage of the n recurrence are much increased [14].

Typically, hypothyroidism is not described in RT, but is rather a gradual finding as the thyroid gland becomes more and more fibrotic. Of our patients five already had hypothyroidism at the time for diagnosis of RT, and one was initially treated with radioiodine because of a toxic adenoma. Antibodies, TPO and thyreoglobulin are often elevated, in up to 90% of patients with RT [6], but if these are markers for the pathogenesis of the disease has been questioned [15]. In this cohort antibodies were present in 3/5 patients. One had all antibodies examined, whereof the elevated TRAb could well be neutral or inhibitory variants, two had antibodies against TPO, one of these also displayed Tgab, and one had only Tgab.

If RT is an IgG4-related disease in all cases, or whether other inflammatory drivers can bring about the same clinical picture is currently unknown. In our cohort only one patient had a clear IgG4/IgG ratio over 40% as has been suggested as a cut off from other forms of IgG4-related diseases [16], however, only three of our cases had enough tissue to have this measured. If all of our patients had RT cannot be totally certain as in case 2 some data are missing, and in case 5 a fibrosing variant of Hashimoto was suggested as an alternative by the pathologist. However, extrathyroidal invasion of inflammatory cells occurred in four cases, as in case 5, and in one the detailed pathology report was missing. Invasion of skeletal muscles, into the parathyroid or in other tissues in the neck are findings consistent with RT [5]. There has been a discussion on the diagnosis and disparities between RT, fibrosing variant of Hashimoto and a third newly described entity IgG4 related Hashimoto (described by Li et al. [17]), where the two latter share some features with RT, but do not involve other organs close to the thyroid [4]. Both the fibrosing variant and IgG4-related Hashimoto are characterized by rapid enlargement of the goiter, and the latter is more common in males. If these two fibrosing diseases are separate or variants of the same disease is unknown, and in the case series from Mayo Clinic there was no difference in IgG4 infiltration between patients with fibrosing variant of Hashimoto´s hypothyroidism and those with RT [12]. The authors speculate that the Hashimoto variant may be an early manifestation that could progress to RT.

IgG4-diseases can affect systemically. In our cohort this was obvious only in case 6. Time of follow-up and diagnostic measures to find and treat other manifestations is important. Previously, other fibrosing variants in organs such as the mediastinum, pancreas, and pseudotumor in the orbit has been described to develop in about a third of patients with RT [11]. One way to find extra thyroid**al** involvement could be the use of FDG-PET, which was not performed in any of our patients. By this modality increased glucose metabolism in active inflammation can be registered, which also can be of value during follow-up [18].

As RT is a rare disease no prospective investigations or randomized control trials have been performed. Based on case reports different treatments have been advocated [5]. Treatment has usually been glucocorticoids [19] with doses up to 100 mg prednisolone daily. A swift response to glucocorticoid treatment is found in most cases, which was also our experience [5, 20]. Recurrence of symptoms is not uncommon and can occur as doses are reduced. Tamoxifen has been used in monotherapy or as an add-on when glucorticoids fail, this can relieve symptoms and achieve goiter reduction, at least together with glucocorticoids [11]. Doses have been between 10 and 20 mg daily. Of our patients four were treated with tamoxifen, of whom two had previously been treated with glucocorticoids. Mycophenolate mofetil was deployed in case 6. This therapy inhibits proliferation of T and B-lymphocytes and antibody production, and has been used previously in a case of RT resistant to glucocorticoids and tamoxifen [21]. In other instances of IgG4-related disease, and resistance to glucocorticoids and tamoxifen, rituximab has been used, with reduction of inflammation symptoms [9]. In the future potential beneficial effects could be speculated in IgG-4 mediated RT resistant to other therapies, by anti-PD1 antibodies such as pembrolizumab or nivolumab since these are monoclonal antibodies type IgG-4κ and IgG-4, respectively.

The inherent limitations of all retrospective studies, above all that of ascertainment bias, was present in this study as well. Moreover, not all biochemical tests, which in hindsight would have been interesting, were done. In addition, not all histological preparations were available to be reviewed.

In conclusion Riedel’s thyroiditis is a rare manifestation within the thyroid gland and adjacent tissues, causing gradual fibrosis in these tissues, leading to pain and hypothyroidism, and in some cases also to hypoparathyroidism and vocal cord palsy. Glucocorticoids are still the mainstay of therapy. Wakefulness of other fibrosing manifestations is important. Immunohistochemistry can show whether plasma cells are increased, and if so IgG4 fibrosis and sclerosis are to be expected in other organs.
